# Antihypertensive and Antidiabetic Drug Candidates from Milkfish (*Chanos chanos*)—Identification and Characterization through an Integrated Bioinformatic Approach

**DOI:** 10.3390/foods13162594

**Published:** 2024-08-19

**Authors:** Roni Nugraha, Fahmi Kurniawan, Asadatun Abdullah, Andreas L. Lopata, Thimo Ruethers

**Affiliations:** 1 Department of Aquatic Product Technology, Faculty of Fisheries and Marine Science, IPB University, Dramaga 16680, Indonesia; aeternoadkurniawan@apps.ipb.ac.id (F.K.); asabdullah@apps.ipb.ac.id (A.A.); 2 Tropical Futures Institute, James Cook University, Singapore 387380, Singapore; andreas.lopata@jcu.edu.au (A.L.L.); thimo.ruethers@jcu.edu.au (T.R.); 3 Molecular Allergy Research Laboratory, Australian Institute of Tropical Health and Medicine, James Cook University, Douglas, QLD 4811, Australia

**Keywords:** ADME analysis, bioactive peptides, in silico, molecular docking, antihypertension, antidiabetic

## Abstract

Integrated bioinformatics tools have created more efficient and robust methods to overcome in vitro challenges and have been widely utilized for the investigation of food proteins and the generation of peptide sequences. This study aimed to analyze the physicochemical properties and bioactivities of novel peptides derived from hydrolyzed milkfish (*Chanos chanos*) protein sequences and to discover their potential angiotensin-converting enzyme (ACE)- and dipeptidyl peptidase-4 (DPPIV)-inhibitory activities using machine learning-based tools, including BIOPEP-UWM, PeptideRanker, and the molecular docking software HADDOCK 2.4. Nine and three peptides were predicted to have ACE- and DPPIV-inhibitory activities, respectively. The DPPIV-inhibitory peptides were predicted to inhibit the compound with no known specific mode. Meanwhile, two tetrapeptides (MVWH and PPPS) were predicted to possess a competitive mode of ACE inhibition by directly binding to the tetra-coordinated Zn ion. Among all nine discovered ACE-inhibitory peptides, only the PPPS peptide satisfied the drug-likeness analysis requirements with no violations of the Lipinski rule of five and should be further investigated in vitro.

## 1. Introduction

Hypertension and diabetes are interconnected comorbidities. The major cause of morbidity and mortality in diabetes is cardiovascular disease, which is exacerbated by hypertension [1]. Hypertension is twice as frequent in patients with diabetes compared with those who do not have diabetes. In 2019, diabetes was the direct cause of 1.5 million deaths, and 48% of all these deaths occurred before the age of 70 years. This problem is amplified by the increasing number of adults with hypertension, predicted to reach 1.56 billion in 2025 [2], and the nearly 7 times higher mortality rate of patients with diabetic hypertension [3]. While there are a few different medications to treat hypertension and diabetes, bioactive peptides have shown promising applications and have been developed as treatments for both conditions.

The growing number of studies of bioactive peptides have attracted the attention of many researchers due to their potential application as therapeutic medications, functional food ingredients, and nutraceuticals [4]. It is known that bioactive peptides exhibit many beneficial activities that can modulate physiological responses, resulting in positive health benefits [5,6]. Many bioactive peptides derived from food sources have been identified as containing antimicrobial, antihypertensive, antioxidant, anticoagulant, antidiabetic, and other beneficial bioactivities [7,8]. Antihypertensive peptides target mainly angiotensin-I-converting enzyme (ACE), which, through the renin–angiotensin system (RAS), plays a crucial role in regulating blood pressure and the electrolyte balance in the human body [9]. Antidiabetic peptides mainly target the dipeptidyl-peptidase 4 (DPPIV) enzyme, which degrades GLP-1, which can, in turn, promote insulin secretion by up to 60% of the total insulin from pancreatic β cells, thereby regulating postprandial blood glucose [10].

Certain procedures are needed to acquire bioactive peptides from food sources; one of them is the hydrolysis of proteins. In discovering bioactive peptides, enzymatic hydrolysis is preferred due to its technical simplicity and environmental friendliness. The enzymatic hydrolysis process is simple, robust, and standardized. The procedure can provide high yields of good-quality bioactive peptides, whereas the biological activities of peptides depend predominantly on their specific structural properties, such as amino acid composition, sequence, chain length, hydrophobicity, and net charge [11].

Recent developments in bioactive peptide research have produced bioinformatics tools to virtually simulate hydrolysis or proteolysis, where it is possible to predict peptide sequences from specific proteins. The advancement of bioinformatics and the enormous number of databases of bioactive peptides have helped to overcome challenges and have been widely utilized for the investigation of proteins and the production of known and novel sequences of peptides from food proteins [12]. The use of these *in silico* tools is time-saving and more efficient than conventional methods and avoids the expense of laboratory time and reagents [6].

Marine-derived food sources such as fish have been extensively studied for sources of bioactive peptides. Research conducted by Abuine et al. [13] shows that fish-skin-derived peptides have a high content of hydrophobic amino acids, which contribute to the antioxidant and angiotensin-converting enzyme-inhibitory activities. Another study [14] discovered bioactivities of peptide sequences from sturgeon skin hydrolysates, such as ACE-inhibitory activity and DPPIV-inhibitory activity. *In silico* studies of bioactive peptides from marine-derived food sources are still emerging and can greatly help in the development of marine-based bioactive products.

Milkfish (*Chanos chanos*) is one of many aquatic organisms with the potential for further bioactive peptide discovery. Milkfish is a brackish-water fish characterized by silver color, cultured in shallow coastal water areas, and capable of living in low-salinity water. A study reported the bioactivity of milkfish collagen peptides, which demonstrated anti-inflammatory activities by reducing lipoxygenase activity and nitric oxide (NO) [15]. Despite several studies describing bioactive peptides from various food sources [16,17,18,19], no study specifically reports the bioactivities of peptides derived from milkfish muscle proteins. The muscle proteins in fish are classified into myofibrillar, sarcoplasmic, and stromal forms, of which myofibrillar proteins make up 50–60% [20]. Myofibrillar proteins, including myosin, actin, tropomyosin, and troponin, are involved in the contraction of the muscle and are attractive substrates to produce hydrolysates due to their rich protein content [21]. Therefore, milkfish muscle protein sequences are compelling for *in silico* studies of bioactive peptides. Molecular docking and computational drug design approaches were used to discover potential ACE-inhibitory and DPPIV-inhibitory peptides.

## 2. Materials and Methods

### 2.1. Protein Extraction and Profiling of Milkfish Muscle Tissue

Muscle tissue samples were taken from the center of the fillet of a whole milkfish specimen and stored at −80 °C until further use. Proteins were extracted in phosphate-buffered saline from both raw and heated tissues, followed by sodium dodecyl sulfate–polyacrylamide gel electrophoresis profiling and the detection of collagen, parvalbumin, and tropomyosin in quantitative immunoblots, as described previously. In brief, proteins were transferred onto a nitrocellulose membrane and, after blocking with casein, incubated with a collagen (ab23730 from Abcam [22]), parvalbumin (in-house-generated [23]), or tropomyosin-specific (in-house-generated [24]) polyclonal antibody, followed by detection with an infrared-labeled antibody (DyLight anti-rabbit 4xPEG by Thermo Scientific, Waltham, MA, USA), visualized with an Odyssey Clx system (LI-COR^®^ Biosciences, Mulgrave, VIC, Australia).

### 2.2. Protein Sequence Selection from the Database

Eight full-length sequences from milkfish muscle proteins (myosin heavy chain, collagen alpha-1, myoglobin, hemoglobin, parvalbumin, troponin T, tropomyosin, calponin, and actin: A0A6J2VHL7, A0A6J2VX00, B9A9V0, A0A6J2WNB3, A0A6J2VB81, A0A6J2WNF1, A0A6J2UQU6, A0A6J2VD67, and A0A6J2VUG0, respectively) were taken from the UniProt (https://www.uniprot.org/ accessed on 6 June 2023) database for *in silico* analysis.

### 2.3. Identification of Protein Physicochemical Properties and Amino Acid Distribution

Protein physicochemical property identification was carried out by using ExPASy’s ProtParam (https://web.expasy.org/protparam/ accessed on 7 June 2023) to determine a large number of physicochemical properties of all the protein sequences identified from milkfish muscle proteins, including the molecular weight, theoretical isoelectric point (pI), total number of negatively charged (Asp + Glu) and positively charged (Arg + Lys) amino acids, total amino acid and atomic composition, extinction coefficient, estimated half-life, and grand average of hydropathicity (GRAVY).

### 2.4. In Silico Proteolysis and Peptide Profiling

*In silico* proteolysis was performed by utilizing BIOPEP-UWM [25] enzyme action tools with three plant proteases, namely, ficin (EC 3.4.22.3), papain (EC 3.4.22.2), and stem bromelain (EC 3.4.22.32). Proteolysis was independently performed based on each protease enzyme. The theoretical hydrolysis degree was also analyzed. The calculation of theoretical hydrolysis used the following equation, where *d* is the number of hydrolyzed peptide bonds, and *D* is the total number of peptide bonds in a protein chain:

$$DHt=\frac{d}{D}\times 100\%$$

The sum of peptides produced from protein proteolysis in BIOPEP-UWM by using the three types of enzymes was considered. The peptides resulting from the enzyme cleavage of each protein by different enzymes were collected, excluding dipeptides and single amino acids. Active peptides released from *in silico* proteolysis by using different enzymes in the BIOPEP-UWM database were further processed by clustering the frequencies of bioactive peptides with specific activities, such as antioxidant, ACE-inhibitory, DPPIV-inhibitory, anti-amnesic, renin-inhibitory, and antithrombotic activities. The frequency of the release of peptides by a specific protease was calculated based on the following equations (AE and W, respectively):

$$A_{E}=\frac{d}{N}$$

‘*d*’ denotes the number of peptides released from the sequence of a protein by a specific protease, and *N* is the number of residues of amino acids present in the protein sequence. The relative frequency of released peptides by specific protease was calculated based on the equations:

$$W=\frac{A_{E}}{A}$$

### 2.5. Novel Peptides’ Bioactivities

Peptides with unknown bioactivity produced from the *in silico* proteolysis process were processed by predicting the bioactivity using PeptideRanker [26] (http://distilldeep.ucd.ie/PeptideRanker/ accessed on 13 June 2023). Predicted peptides with a threshold above or equal to 0.7 were considered as potentially bioactive peptides. Peptides that have a value of less than 0.7 were discarded. Bioactivity screening was performed by using MultiPep [27] (https://agbg.shinyapps.io/MultiPep/ accessed on 24 June 2023) to predict the specific bioactivities of peptides. MultiPep utilizes a machine learning approach to rank peptides based on their specific bioactivity. Peptides with a specific activity value of 0.5 or more can be considered as potential bioactive according to the specific activity.

### 2.6. Peptide Structure Modeling and Docking

Peptides with five or more residues were predicted by using Alphafold [28], while tetrapeptides were designed by using Discovery Studio Visualizer 3.0 software (Accelrys Software, Cambridge, UK). The crystallographic structure of human ACE-I (PDB ID: 1O86) was obtained from the Protein DataBank (https://www.rcsb.org/ accessed 18 July 2023). The structure of the DPP-IV enzyme complex with piperidine-constrained phenethylamine was obtained from the PDB (PDB ID: 2OQV). Both receptors were prepared by removing the water molecules and bound ligands from the active site residues. The peptide’s active sites were predicted by Pepsite 2 [29] (http://pepsite2.russelllab.org/ accessed on 18 July 2023).

Molecular docking was performed by using the HADDOCK 2.4 cloud server [30]. The docking of peptides with the prepared receptor was performed separately by using default parameters for protein and peptide interactions. The active residues of the receptors were obtained from the literature, while the active residues of the designed peptides were found by using Pepsite 2 (http://pepsite2.russelllab.org/ accessed on 18 July 2023). The passive residue area of the receptors was set to 5 Å from the active residue. Clusters produced from the docking process with the best HADDOCK score and a root-mean-square deviation (RMSD) less than 4 Å were further evaluated for the best pose. The best pose was discovered by evaluating the molecular interactions between the peptides and binding sites in each cluster. HADDOCK scoring uses the sum of terms whose weights depend on the stage of the HADDOCK protocol:

$$\begin{array}{c} it0:\;0.01EvdW+0.1Eelec+1.0Edesolv–0.01BSA+0.01EAIR\\ it1:\;1.0EvdW+1.0Eelec+1.0Edesolv–0.01BSA+0.1EAIR\\ itw:1.0EvdW+1.0Eelec+1.0Edesolv+0.01EAIR \end{array}$$

The binding affinity was subsequently predicted from the selected best pose by entering the model into protein binding energy prediction tools (PRODIGY) [31]. The protein–peptide binding affinity was predicted by using the following equation:

$$\begin{array}{cc} \Delta Gpredicted= & -0.09459\frac{ICscharged}{charged}-0.10007\frac{ICscharged}{apolar}\\ & +0.19577\frac{ICspolar}{polar}-0.22671\frac{ICspolar}{apolar}\\ & +0.18681\%NISapolar+0.3810\%NIScharged-15.9433 \end{array}$$

ICs stands for interfacial contacts in a protein–protein complex and NIS for non-interacting surfaces. The dissociation constant was calculated by using the Gibbs free energy based on the following equation:

ΔG=RT lnKd

where R is the ideal gas constant (in kcal K^−1^mol^−1^), T is the temperature (in K), and ΔG is the predicted free energy. By default, the temperature is set at 298.15 K (25.0 °C). The interactions between the peptides and the binding sites were evaluated by using Maestro software version 2023-2 (Schrödinger, LLC, New York, NY, USA, 2023) using a two-dimensional ligand–receptor interaction map with an area of 4 Å surrounding the peptide. A drug-likeness analysis of the peptides was performed with the ADMETlab 2.0 cloud server [32] (https://admetmesh.scbdd.com/ accessed on 29 August 2023) as a systematic evaluation of ADMET properties, as well as some physiochemical properties and medicinal chemistry friendliness.

## 3. Results

### 3.1. Protein Profiling and Identification

**Figure 1:**
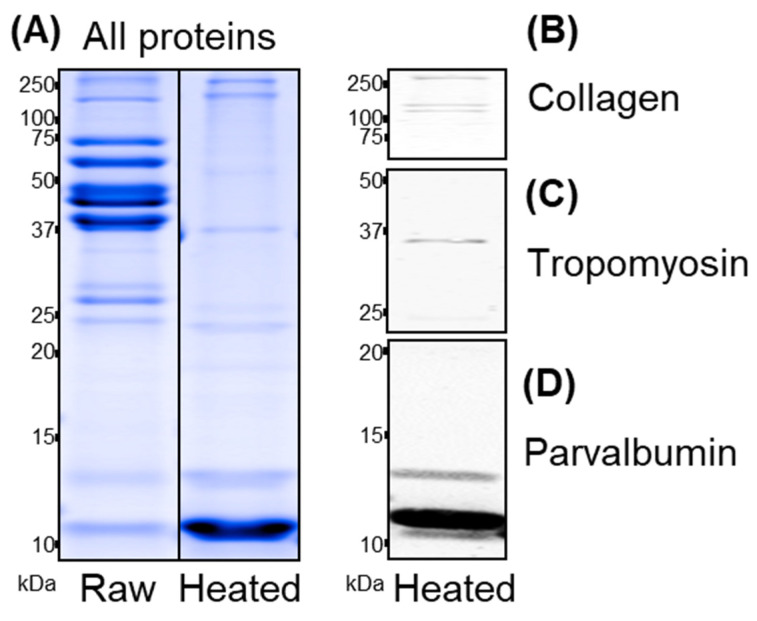
Coomassie-stained total protein profiles of raw and heated milkfish muscle extracts (**A**) and detection of collagen (**B**), tropomyosin (**C**), and parvalbumin (**D**) in the latter utilizing antibodies in immunoblotting analyses.

The total protein profiles of raw and heated milkfish muscle extracts were analyzed and are shown in Figure 1. Bands at 10–12 kDa are the most prominent in both raw and heated extracts and consist of parvalbumin, as demonstrated by immunoblotting. In heated extracts, collagen at ≥100 kDa and tropomyosin at 35 kDa were identified by immunoblotting, which are the most abundant soluble and heat-stable muscle proteins after parvalbumin, as also previously demonstrated for catfish and salmon, suggesting high yields after hydrolysis [33].

### 3.2. Protein Physicochemical Properties and Amino Acid Distribution

The FASTA sequences of nine proteins obtained from the UNIPROT database were analyzed. Expasy’s Protparam tool was used to compute the parameters to analyze the physicochemical properties of the retrieved proteins. Parameters such as negatively charged residues, positively charged residues, instability index, aliphatic index, grand average of hydropathy (GRAVY), and molecular weight were calculated. The results are shown in Table 1.

Of the nine analyzed proteins, most contain a higher number of negatively charged residues compared to positively charged residues, ranging from 50 to 277 residues, and only one protein (myoglobin) showed a higher number of positively charged residues. The instability index ranged from 10 to 46, with the highest value found in tropomyosin and the lowest found in myoglobin. The aliphatic index ranged from 49 to 102, with the highest value found in myoglobin and the lowest found in collagen alpha 1. The grand average of hydropathy value of the nine proteins ranged from −1 to 0, and the heaviest molecular weight was found in the myosin heavy chain.

Charged residues in a protein are known to confer the specificity of interactions, as opposite charges attract, whereas like charges repel each other. The interactions of charged residues with polar groups, particularly in the form of hydrogen bonds, reinforce interaction specificity, whereas charge–charge interactions can be strong even at a distance (e.g., of 5–10 Å) [34]. Such interactions may have a role in determining the constant rate of protein binding with its ligand or macromolecular counterpart [35]. The instability index is used to predict whether the proteins can be considered stable or unstable. Proteins with an instability index value of over 40 are considered unstable [36]. From the data above, it is known that only four proteins can be considered stable (hemoglobin, myoglobin, parvalbumin, and actin). The instability index is correlated with the in vivo half-life of the protein molecules. Proteins that have an in vivo half-life of less than 5 h have been shown to have an instability index of more than 40, whereas those that have an in vivo half-life of more than 16 h have an instability index of less than 40 [37]. The stability of proteins during the process of expression and purification (experiments) is one of the crucial and challenging issues because many recombinant proteins are unstable under the conditions in which they are expressed and lose their correct folding or undergo proteolytic digestion [38].

The aliphatic index can be defined as the relative volume of a protein occupied by its aliphatic side chains. A higher aliphatic index indicates that the proteins are more thermally stable over a wide temperature range, and it is also noted that aliphatic amino acids are hydrophobic in nature [39,40]. In this study, the aliphatic index of proteins ranges from 49 to 102, where the lowest can be found in collagen alpha 1. Collagen is known to be highly stable at high temperatures [22], but its aliphatic index tended to be the lowest among the nine proteins of interest in this study.

The low value of the aliphatic index for collagen alpha-1 is attributed to the low number of aliphatic amino acids in the sequence. In the case of collagen’s thermostability, it should be considered that the hydroxyproline in collagen plays a significant role in thermal stability [41,42]. An increased hydroxyproline (Hyp) content in the collagen structure model was beneficial in improving the thermal resistance of the structure. Thermal unfolding did not occur simultaneously along the entire molecule but started in the regions with lower Hyp content, as the Hyp residue can create additional hydrogen bonds between collagen chains to increase the thermal stability of collagen molecules [43].

The hypothetical and conserved proteins in this study had GRAVY indexes ranging from −1 to 0.095. This low GRAVY range indicates the possibility of being a globular (hydrophobic) protein rather than membranous (hydrophilic). GRAVY values in the range of −2 to +2 are indicative of the proteins being more hydrophobic [36]. Positive GRAVY values indicate hydrophobicity, while negative values mean hydrophilicity [44]. Myosin heavy chain is the heaviest protein, with a molecular weight (MW) of 182 kDa, while parvalbumin is the lightest, with a molecular weight of 11 kDa. The MW of a protein can be calculated based on its amino acid (AA) composition [45]. The longer the amino acid sequence of a protein, the higher the molecular weight.

The amino acid distribution was also analyzed using Expasy’s Protparam tool. Twenty amino acids were evaluated in each protein; each amino acid was clustered based on its properties, such as negatively charged (glutamic acid and aspartic acid), positively charged (lysine and arginine), non-polar (valine, proline, methionine, leucine, isoleucine, glycine, and alanine), polar (threonine, serine, glutamine, asparagine, histidine, and cysteine), and aromatic amino acids (tyrosine, tryptophan, and phenylalanine). The properties of amino acids are important for the bioactivities of peptides, as the composition and sequence determine the activities of the peptides once they are released from the precursor protein in which they are incorporated [46].

### 3.3. Peptide Frequency

**Figure 2:**
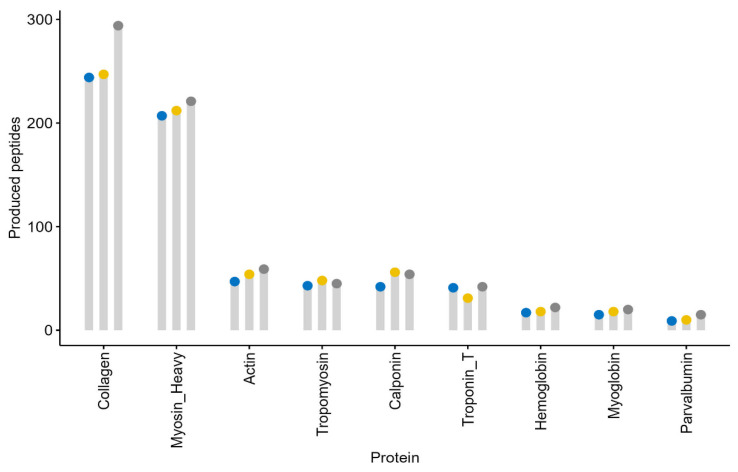
A dot chart of the peptide frequency after enzymatic hydrolysis. The peptide frequency was calculated from all possible peptides generated after the hydrolysis of proteins using bromelain (blue dots), ficin (yellow dots), and papain (gray dots). Only peptides with more than two amino acids were considered.

The number of peptides generated was observed. The criteria for summed peptides were tripeptides and above, while dipeptides were excluded. The produced peptides were collected from virtual protein cleavage and are visualized in a dot chart in Figure 2.

**Figure 3:**
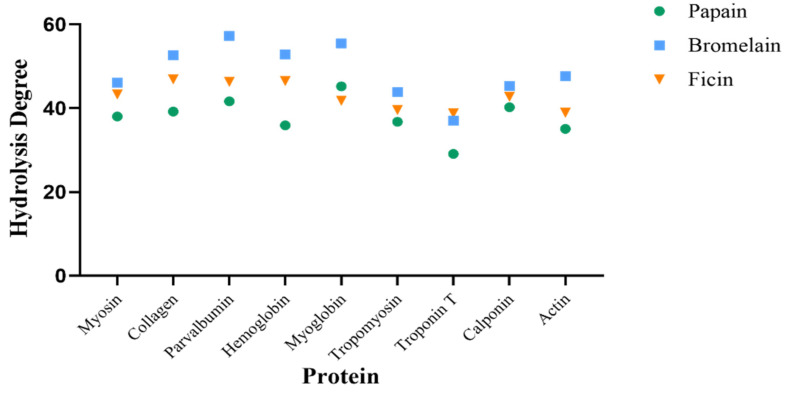
A hydrolysis degree comparison. The hydrolysis degrees of the proteins after hydrolysis by papain, bromelain, and ficin are represented by the green circles, blue squares, and orange triangles, respectively.

The number of produced peptides ranges from 9 to 294, with papain producing the highest number of peptides among the nine selected proteins. The production of peptides is correlated with the hydrolysis degree of the proteolytic process, as an enzymatic proteolysis process is often quantified as the degree of hydrolysis (DH), which represents the percentage of peptide bonds cleaved compared to the initial number of peptide bonds of the protein [47]. A higher hydrolysis degree results in more peptide bonds being cleaved by the enzyme. DH comparisons are visualized in Figure 3.

The DH ranges from 29 percent as the lowest to 57 percent as the highest. A higher hydrolysis degree results in the generation of more peptides by the enzyme. The highest number of peptides is known to be from the papain enzyme, but the hydrolysis degree of papain tends to be lower compared to the other two enzymes (see Figure 3). Such phenomena can be attributed to the criteria used for the sum of produced peptides. Bromelain and ficin may produce more dipeptides or even single amino acid residues than papain through enzyme cleavage, but single amino acid residues and dipeptides were excluded from the analysis. The degree of hydrolysis can be defined as how much the protein is hydrolyzed and is measured by the number of peptide bonds cut, which is then divided by the total number of peptide bonds in a protein and multiplied by 100 [48]. Hence, the higher the hydrolysis degree is, the shorter the produced peptides tend to be.

### 3.4. Profiling of Bioactive Peptides

The bioactive peptides produced from enzyme cleavage were observed. Bioactivities such as antioxidative, ACE-inhibitory, DPPIV-inhibitory, anti-amnesia, and renin-inhibitory activities were identified from the nine proteolyzed proteins. The bioactive peptides were automatically identified from the BIOPEP-UWM database after the proteolysis process had occurred. The overall bioactivity potentials (∑A_E_) of the peptides released from the proteins after enzymatic hydrolysis are shown in Table 2.

The AE values of six bioactivities from three enzymes are the highest for ACE-inhibitory and DPPIV-inhibitory activities. The result of released bioactive peptides from milkfish proteins is also supported by the number of studies regarding the ACE- and DPPIV-inhibitory activities from fish or marine organisms. A study conducted by Hong et al. [49] successfully discovered two peptides extracted from the silver carp swim bladder with good inhibition of soluble DPP-IV and insulin secretion promotion. A review study also showed the potential of ACE-inhibitory biopeptides extracted from several fish species [50]. Meat and fish proteins offer considerable potential as novel sources of bioactive peptides, as many of the studies conducted to date have focused on the production and identification of DPP-IV-inhibitory and ACE-inhibitory peptides from protein hydrolysates from different food systems [20,51].

### 3.5. Novel Peptide Bioactivity Screening

Peptides from the virtual proteolytic process with the three enzymes whose bioactivities were not identified by the database were collected and evaluated by PeptideRanker and MultiPep for their potential bioactivities. From the 2132 peptides collected in this study, 75 peptides with a threshold score for general bioactivities of more than or equal to 0.7 were identified by PeptideRanker. The 75 peptides were further evaluated for their ACE- and DPPIV-inhibitory activities by using MultiPep. Twelve novel peptides with promising ACE- and DPPIV-inhibitory activities were identified and are listed in Table 3.

Of all evaluated peptides, seven peptides are known to specifically exhibit ACE-inhibitory activity, and one peptide specifically exhibits DPPIV-inhibitory activity, while some peptides only fulfill criteria for being antihypertensive or antidiabetic. Nevertheless, these peptides (PMIPG, YPPPT, AAWMIY, and AWMIYT) were also tested in this study, regardless of their low scores, to evaluate their possible ACE- or DPPIV-inhibitory activity. The terms antihypertensive and antidiabetic can refer to mechanisms of action other than ACE and DPPIV inhibition. The antihypertensive peptides in Table 3 may involve mechanisms of action such as renin inhibition, calcium channel blocking, angiotensin II receptor blockers (ARBs), etc. [9]. Meanwhile, the antidiabetic peptides may involve mechanisms such as α-amylase or α-glucosidase inhibition [52].

### 3.6. Molecular Docking Analysis

The twelve potential peptides from the previous analysis were collected for molecular docking modeling. Prior to the molecular docking process, each peptide structure was modeled by using two different applications. Pentapeptides and above were designed by using Alphafold 2, while the smaller tetrapeptides were designed by using Discovery Studio. Molecular docking was carried out by using HADDOCK 2.4 separately, as multi-ligand docking is not recommended for the docking tool used. The docking result is shown in Table 4.

The HADDOCK scores obtained range from −97 to −50; RMSD ranges from 0 to 1, and the values of Z-scores are less than one. The HADDOCK score value cannot directly imply whether the docking result is successful or not but rather determines the best clusters from the molecular docking process. The RMSD value indicates the average distance between the best four models of the specified cluster and the best-scoring model generated by HADDOCK. It provides information about how much the best four models of specified clusters deviate compared to the best-scoring model. The *z*-score represents how many standard deviations by which the HADDOCK score of a given cluster is separated from the mean of all clusters, meaning the lower the *z*-score, the better [30]. The Z-score corresponds to the HADDOCK score, as the lowest HADDOCK score means the lowest Z-score obtained. Overall, the selection of the best cluster from the docking process by using HADDOCK should mainly be based on the HADDOCK score itself. Further validation, such as binding affinity prediction, can also be performed to enhance the docking prediction.

The binding affinity of docked peptides according to HADDOCK was predicted by using the PRODIGY tool. The binding affinity can be defined as the strength of the interaction between the receptor and the ligand and can also be translated into physicochemical terms as the dissociation constant (Kd), which is an experimental measure that determines whether an interaction will occur in solution or not [53]. The binding affinity predictions for the novel peptides are shown in Table 5.

The binding affinities of all studied peptides range from −12.3 to −8.5 kcal/mol. The binding affinity prediction is designed to provide the most accurate estimate of the strength with which a molecule binds to a macromolecular target [54]. Hence, a lower value of binding affinity means that a more stable complex is formed [55]. However, a molecular interaction analysis of the complexes should be performed to see whether the interaction targets the specified active sites of both ACE and DPPIV.

### 3.7. ACE-Inhibitor Molecular Interaction

**Figure 4:**
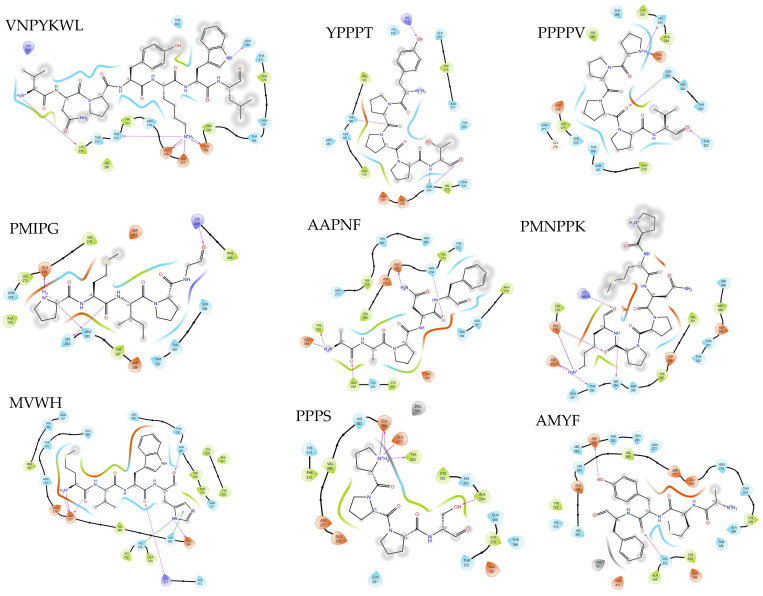
Two-dimensional interaction diagrams of peptides exhibiting ACE-inhibitory activity in the active sites of angiotensin-converting enzyme. Residues are represented in different colors, with blue for polar amino acids, green for hydrophobic amino acids, orange for negatively charged amino acids, purple for positively charged amino acids, and gray for metal ions. The purple arrow line and straight blue-red line represent hydrogen bond and salt bridge interactions.

The nine discovered peptides have been demonstrated to exhibit ACE-inhibitory activity; six peptides consisted of 5 to 7 residues (VNPYKWL, PMNPPK, PPPPV, PMIPG, YPPPT, and AAPNF), and the other three peptides are classified as tetrapeptides (AMYF, MVWH, and PPPS). The docked peptides need to be further analyzed by evaluating the interacting residues on the receptor. The receptor was derived from the crystal structure of the human angiotensin-converting enzyme, which was obtained from the PDB (PDB id: 1O86) and composed of 589 amino acids sequences. This enzyme is classified as a metalloprotease and is also well known for its dual actions in converting inactive Ang I to active Ang II, which plays an important role in the control of blood pressure [56]. As a metalloprotease, the zinc ion in ACE plays a vital role in the catalytic process. ACE has three active pockets: S1 (Ala 354, Glu 384, and Tyr 523), S2 (Gln 281, His 353, Lys 511, His 513, and Tyr 520), and S1′ (Glu 162) [57]. The molecular interactions between each peptide and the receptor play an important role in the ACE-inhibitory activity of the peptides, as more interactions with the ACE active sites may result in potent activity against ACE. The two-dimensional protein–peptide interaction diagrams are visualized in Figure 4. The molecular interactions observed in Figure 4 are non-covalent, such as hydrogen bonds (purple-colored lines), salt bridge interactions (red-blue colored lines), and pi stacking (green-colored lines). Interactions at active sites play an important role in the inhibition of ACE, as they may disrupt the catalytic activity. The interacting residues of nine ACE-inhibitory peptides are displayed in Table 6.

All docked ACE-inhibitory peptides interact with active sites located within the pockets (S1, S2, and S1′), with the exception of two peptides (PMIPG and PMNPPK). Peptides composed of five or more residues only interact with a few active sites, and some (PMIPG and PMNPPK) do not show any interactions with the given active sites. The peptide AAPNF establishes two hydrogen bonds and one salt bridge interaction with three active sites in S1: Ala 354, Tyr 523, and Glu 284, respectively. The other peptides, such as VNPYKWL, YPPT, and PPPPV, also display hydrogen bond interactions with active residues, but only VNPYKWL and PPPPV also display salt bridge interactions with the same residue (GLU 162).

It should be noted that both hydrogen bonds and salt bridges play a role in binding stabilization. Hydrogen bond interaction forces play the most important role in stabilizing the docking complex and enzyme catalytic reaction [58]. The salt bridge interaction, on the other hand, is the strongest non-covalent interaction in nature and is known to participate in protein folding, protein–protein interactions, and molecular recognition [59]. The mentioned peptides (VNPYKWL, YPPT, PPPPV, AAPNF) may exhibit good inhibitory effects against ACE based on the interaction analysis.

Of all predicted ACE-inhibitory peptides, the two peptides PMIPG and YPPPT do not meet the threshold score, yet YPPPT shows interaction with the active site (Lys 511), while PMIPG does not show any interaction. The MultiPep tool utilizes convolutional neural networks to predict the peptide class to which a peptide belongs and can classify peptides into zero or more bioactivity classes based on their intrinsic amino acid patterns [27]. It is possible that the system could not recognize the patterns of both peptides to be ACE-inhibitory. Additionally, the value is probabilistic rather than binary; therefore, such phenomena may be expected. Predictions below the threshold might still indicate that given peptides have properties associated with a certain class [27].

Another interesting case was found with PMNPPK. The peptide PMNPPK possesses the highest ACE-inhibitory score, but it does not show any interactions with known active sites. A similar finding was reported in a study [60] on trypsin hydrolysates of salmon bone proteins, where a peptide (FCLYELAR) with ACE-inhibitory activity did not interact with any active sites (S1, S2, S1′) of ACE during molecular docking simulations. The study suggested that the peptide exhibits an uncompetitive mode of inhibition. Therefore, based on these findings, the peptides PMNPPK and PMIPG may possess a similar mode of inhibition. However, an in vitro assay is required to validate this claim.

In contrast, the tetrapeptides (MVWH, AMYF, and PPPS) show far more satisfactory results. The MVWH and PPPS peptides interacted with the tetra-coordinated zinc ions of two residues (His 383 and Glu 411). The zinc ion has a tetra-coordinate formation with three ACE residues (His 383, His 387, Glu 411), where the distortion or disruption of the tetrahedral geometry can cause ACE-inhibitory activity [61]. By directly interacting with tetra-coordinated zinc, the peptides MVWH and PPPS have a higher probability of exhibiting a competitive mode of inhibition. The relationships between ACE-inhibitory activity and peptide structure have not been fully elucidated; it is possible to conclude that the inhibitory potential of the peptide depends on its structural and compositional characteristics [62]. It is suggested that hydrophobic, branched-chain, or aromatic amino acids are important components of ACE-inhibitory peptides, as they would be compatible with the ACE active site [19,63]. The amino acid composition as a whole seems only to affect the smaller peptides, while the inhibitory effect of peptides with longer residues has been related to C-terminal amino acids [62,64].

### 3.8. DPPIV-Inhibitor Molecular Interaction

Three potential peptides with DPPIV-inhibitory activity were discovered through virtual screening. Two peptides are composed of six amino acid residues (AWMIYT and AAWMIY), and one peptide is a tetrapeptide (MQML). The receptor is based on the crystal structure of Human Dipeptidyl Peptidase IV (DPP4) (PDB ID: 2OQV). DPPIV is classified as a serine protease with a serine, histidine, and aspartic acid catalytic triad of amino acids and has the potential to cleave peptide bonds to form a penultimate proline residue and release proline-containing dipeptides from the N-terminus of the polypeptide chain [65].

**Figure 5:**
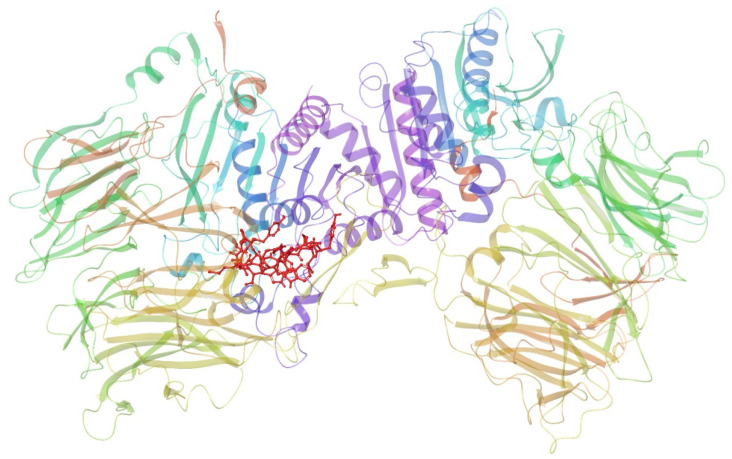
DPPIV-inhibitory peptide interaction with DPPIV chain A (PDB ID: 2OQV). The red-colored structure represents the conformation of the peptides inside the active site of DPPIV.

DPPIV has three binding pockets/active sites (S1, S2, and S3) [66,67,68]. S1 consists of Tyr 547, Ser 630, Tyr 631, Val 656, Trp 659, Tyr 662, Tyr 666, Asn 710, Val 711, and His 740, all of which are involved in strong hydrophobic interactions. The residues Ser 630, Asp 708, and His 740 form the catalytic triad [69]. S2 is the cavity near Arg 125, Glu 205, Glu 206, and Tyr 662. S3 consists of Ser 209, Arg 358, and Phe 357. The interaction of DPPIV-inhibitory peptides with DPPIV is shown in Figure 5.

**Figure 6:**
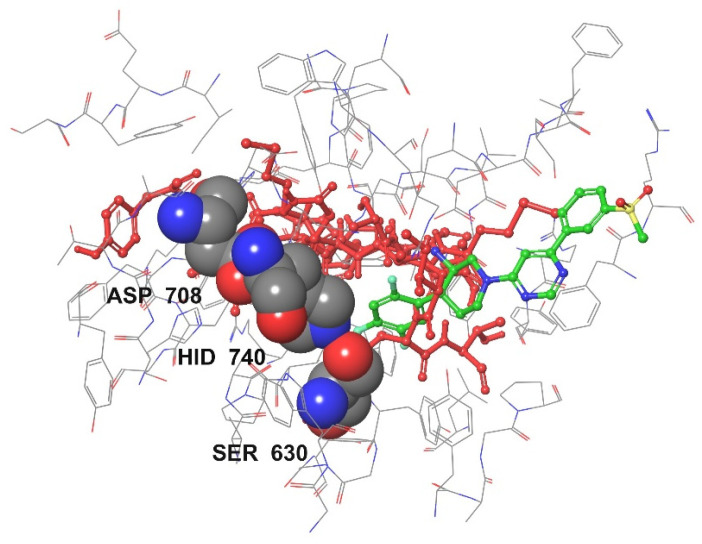
DPPIV-inhibitory peptides and piperidine-constrained phenethylamine interaction with DPPIV. The tube structure models represent the amino acids of DPPIV at active sites, while the ball-and-stick model represents the DPPIV-inhibitory peptides, and the space-filling model represents the phenethylamine structure.

Figure 5 shows the conformation of all three DPPIV-inhibitory peptides in DPPIV. The red-colored backbones represent the conformation of the peptides inside the DPPIV pocket sites. The molecular interaction takes place in chain A of DPPIV, and chain B of DPPIV is identical to chain A. The peptide conformation is also compared to piperidine-constrained phenethylamine (green-colored compound), a potent and selective DPPIV inhibitor, and is shown in Figure 6.

**Figure 7:**
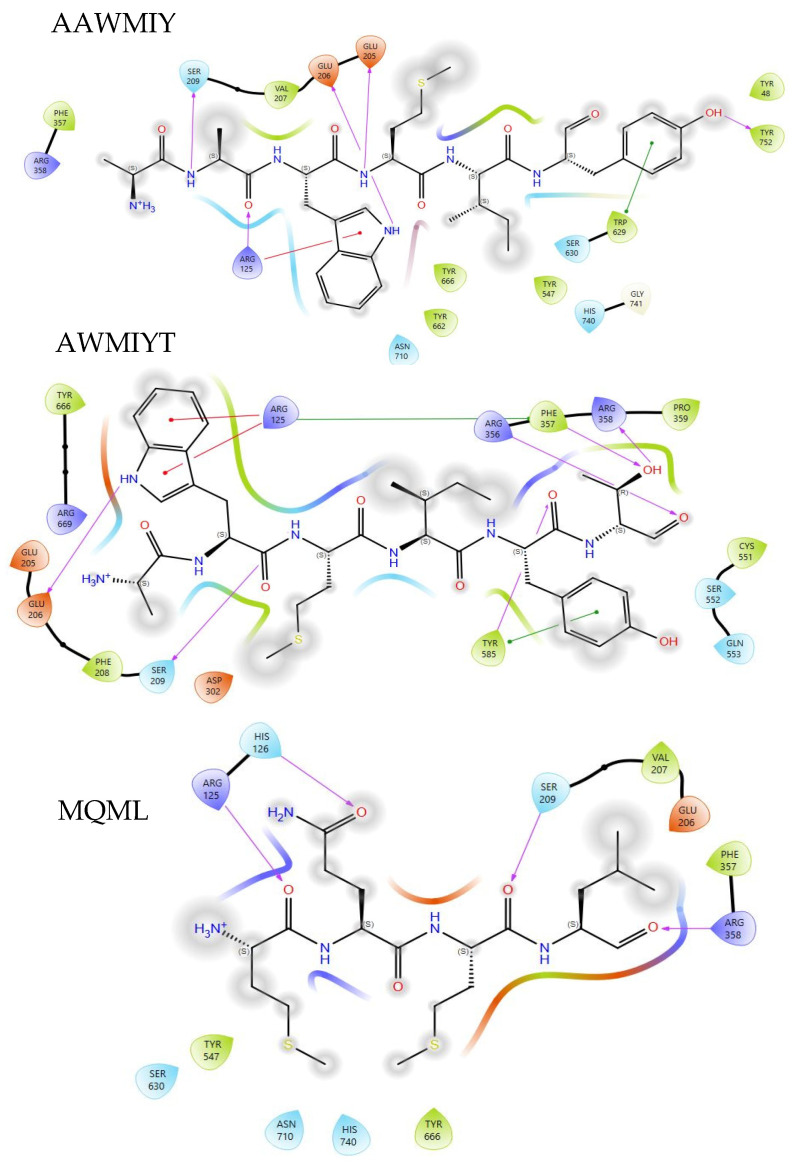
Two-dimensional interaction diagrams of AAWMIY, AWMIYT, and MQML. Residues are represented in different colors, with blue for polar amino acids, green for hydrophobic amino acids, orange for negatively charged amino acids, purple for positively charged amino acids, and gray for metal ions. The purple arrow line, green line, and straight blue-red line represent hydrogen bond, Pi-Pi stacking, and salt bridge interactions.

The peptides bind to the active sites in the cavity, as shown in the two visualizations above. The globular shapes in Figure 6 represent the catalytic triad (SER 630, ASP 708, and HIS 740) of DPPIV. These peptides have the potential to exhibit satisfactory DPPIV inhibition activity, as they interact with and bind to the active sites. Two-dimensional protein–peptide interaction diagrams are visualized in Figure 7.

The molecular interactions observed in Figure 7 consist of non-covalent interactions such as hydrogen bonds (purple-colored lines), pi–cation interactions (red-colored lines), and Pi stacking (green-colored lines). The interacting residues of ACE-inhibitory peptides are displayed in Table 7. The DPPIV-inhibitory peptides interact with the active sites of DPPIV in S2 and S3, but none interact with S1 residues. Two peptides (AAWMIY and AWMIYT) interact with Glu 206 and Arg 125, while MQML only interacts with Arg 125, in common with the other two peptides. The residues Arg 125, Glu 205, Glu 206, Tyr 547, Tyr 662, and Tyr 666 are key amino acid residues in ligand and receptor interactions [70]. Recent studies suggest that hydrophobic interactions in the S1 pocket are crucial for DPPIV-inhibitory peptides, and the interaction at the S2 pocket may improve affinity [71]. Another study considered competitive inhibitory peptides that were predicted to have both hydrophobic and hydrogen bond interactions with the active site of DPPIV [72]. Nevertheless, it has been reported that different peptides show different DPPIV-inhibitory modes, such as competitive, uncompetitive, non-competitive, and mixed-type modes [73]. With high probability, the three peptides (AAWMIY, AWMIYT, and MQML) might exert DPPIV-inhibitory activities by binding either at the active site and/or outside the catalytic site of DPPIV. 

### 3.9. Drug-Likeness Analysis

The concept of drug-likeness is established from analyses of the physiochemical properties and structural features of existing small organic drugs or drug candidates. This has been widely used to remove compounds with undesirable properties, especially those with poor ADMET (absorption, distribution, metabolism, excretion, and toxicity) profiles [74]. Pre-clinical and clinical trials are time-consuming and responsible for most of the drug development costs. Hence, the drug-likeness of compounds should be determined as early as possible in the design process for cost and time efficiency. The predicted absorption, distribution, and toxicity of the peptides are shown in Table 8.

Madin−Darby canine kidney cells (MDCK) have been developed as an in vitro model for permeability screening and are widely considered to be the in vitro gold standard for assessing the uptake efficiency of chemicals by the body. The unit of predicted MDCK permeability is cm/s. A compound is considered to have a high passive MDCK permeability if Papp > 20 × 10^−6^ cm/s, medium permeability if 2–20 × 10^−6^ cm/s, and low permeability if <2 × 10^−6^ cm/s. Four peptides are predicted to have high passive permeability (VNPYKWL, AAPNF, YPPPT, and PPPS), two peptides to have poor permeability (PMIPG and MVWH), and the other peptides to have medium permeability (PMNPPK, PPPPV, AMYF, AAWMIY, and AWMIYT). Of all of these peptides, four (MVWH, AAWMIY, AWMIYT, and MQML) are predicted to have good intestinal absorbability. All peptides fulfill the optimal distribution parameters (a VD in the range of 0.04–20 L/kg and plasma protein binding not exceeding 90%). As for toxicity, most peptides are predicted to be hepatotoxic, except AAPNF, MVWH, and MQML. All of the screened peptides are also predicted not to be genotoxic or able to induce mutations in cells.

The specific physiochemical properties of the peptides were also evaluated to establish compliance with orally administered drug-likeness guidelines known as the Lipinski rule of five (ROF). The rule of five predicts that poor absorption or permeation is likely to occur when there are more than five hydrogen bond donors and ten hydrogen bond acceptors, the molecular weight is greater than 500, and the calculated log P (log P) is lower than five [75]. The physicochemical and physiochemical properties of the identified peptides are shown in Table 9. The simple physicochemical properties of molecules, such as molecular weight (MW), the number of hydrogen bond donors (HBDs) and acceptors (HBAs), hydrophobicity, and the polar surface area (TPSA), can affect their in vivo behavior and influence their efficiency in molecular targeting [76]. Another factor, the octanol/water partition coefficient (log P), greatly affects the lipophilicity of a compound [77]. It should be noted that highly lipophilic compounds can be trapped in the bilayer due to their poor penetration of membranes, as high lipophilicity and poor aqueous solubility cause the inability of small compounds to solubilize completely in aqueous media [78]. Of all the evaluated peptides, only one peptide complies with the Lipinski rules (PPPS). This peptide was derived from milkfish collagen. This peptide has previously been reported to bind the active site of dipeptidyl carboxypeptidase derived from Streptomyces [79]. This enzyme is analogous to angiotensin-I-converting enzyme (ACE), which plays a critical role in the regulation of blood pressure homeostasis. The findings of this study corroborate current results, demonstrating that integrated bioinformatic techniques can effectively identify potential drug candidates.

Compounds violating more than two of the RO5 conditions are prone to cause gastrointestinal absorption problems [80]. This has always been an issue in peptide-based drug development, as the use of peptides in therapy presents several limitations, from physiochemical characteristics to inadequate pharmacokinetic profiles for oral absorption [81]. Nevertheless, peptide drug development has made great progress in the last decade due to production, modification, and analytic technologies, where peptides have been produced and modified using both chemical and biological methods [82].

## 4. Conclusions

Conventional methods for identifying and characterizing bioactive peptides often involve extensive laboratory analyses, which are time-consuming, costly, and labor-intensive. Furthermore, these techniques may overlook low-abundance peptides and require considerable expertise, potentially limiting the discovery of novel peptides with therapeutic potential and delaying the development of new treatments. Integrated bioinformatics methods for the identification of bioactive peptides offer several advantages, including speed, cost-effectiveness, and the ability to analyze large datasets rapidly. Peptide activity, stability, and interactions can be predicted with high accuracy using computational approaches, reducing the need for extensive laboratory work. In addition, integrated bioinformatics tools allow the screening of numerous peptide sequences simultaneously, facilitating the efficient discovery of novel peptides with therapeutic potential.

In this study, nine stable and abundant milkfish muscle proteins were selected and hydrolyzed using three different proteases, generating over 2000 peptides *in silico*. The peptide pool was rigorously screened using an integrated bioinformatics approach involving BIOPEP-UWM, PeptideRanker, and HADDOCK 2.4 to predict bioactivities. A drug-likeness analysis was performed with ADMETlab to evaluate ADMET properties, physicochemical characteristics, and medicinal chemistry suitability. This workflow yielded several peptides with high ACE- and DPPIV-inhibitory activities, as well as satisfactory scores and favorable interactions with the receptors’ defined active sites. Two ACE-inhibitory tetrapeptides (MVWH and PPPS) were predicted to possess the competitive mode of ACE inhibition by directly binding to the tetra-coordinated Zn ion. Three peptides were found to inhibit DPPIV with unspecific modes. The drug-likeness analysis resulted in one peptide (PPPS), derived from high-abundance and heat-stable milkfish collagen, that satisfied the Lipinski rule of five and has the potential to be an orally administered ACE-inhibitory drug candidate. While molecular docking analyses provided insights into potential interactions, experimental validation through in vitro or in vivo assays is necessary to confirm the bioactivity, bioavailability, and therapeutic potential of the identified peptides.

## Figures and Tables

**Table 1:** Physicochemical properties of proteins selected for this study.

Protein	^a^ −R (Asp + Glu)	^b^ +R (Arg + Lys)	Instability Index	Aliphatic Index	^c^ GRAVY	Molecular Weight (Da)	Accession ID
Myosin heavy chain	277	47	44.15	81.96	−0.646	182,253.3	A0A6J2VD67
Collagen alpha-1	195	60	40.81	49.3	−0.778	180,196.21	A0A6J2VX00
Hemoglobin	14	7	25.69	90.07	−0.044	15,644.13	A0A6J2WNB3
Myoglobin	15	17	10.36	102.31	0.095	15,667.24	B9A9V0
Tropomyosin	79	4	45.54	81.9	−1.032	32,984.17	A0A6J2VHL7
Troponin T	76	65	85.2	58.93	−1.525	33,986.17	A0A6J2VB81
Parvalbumin	20	13	27.35	79.91	−0.043	11,613.04	A0A6J2UQU6
Calponin	36	34	41.9	61.77	−0.728	34,072.12	A0A6J2WNF1
Actin	50	37	34.36	82.81	−0.221	41,974.92	A0A6J2VUG0

^a^ −R is the symbol for negatively charged residues, ^b^ +R is the symbol for positively charged residues, ^c^ GRAVY is an abbreviation for the grand average of hydropathy.

**Table 2:** Released bioactive peptides by three different enzymes.

Enzyme	∑A_E_					
	Antioxidative	ACE Inhibitor	DPPIV Inhibitor	Anti-Amnesia	Renin Inhibitor	Antithrombotic
Papain	0.0343	0.5563	0.667	0.0237	0.0211	0.0231
Bromelain	0.033	0.5585	0.5983	0.0484	0.0377	0.0484
Ficin	0.0694	2.2332	3.2131	0.0371	0.0644	0.0365

∑A_E_: The frequency of the release of fragments with a given activity.

**Table 3:** Novel peptides’ specific bioactivity scores.

No.	Peptide	ACE-Inhibitory Score	Antihypertensive Score	Antidiabetic Score	DPPIV-Inhibitory Score
1	PMNPPK	0.977	−	−	−
2	VNPYKWL	0.663	−	−	−
3	AAPNF	0.51	−	−	−
4	PPPPV	0.582	−	−	−
5	PMIPG	−	0.818	−	−
6	YPPPT	−	0.837	−	−
7	AMYF	0.528	0.903	−	−
8	PPPS	0.591	0.957	−	−
9	MVWH	0.612	0.936	−	−
10	AAWMIY	−	0.778	0.574	−
11	AWMIYT	−	0.517	0.519	−
12	MQML	−	−	0.813	0.594

(−) denotes scores below the threshold (0.5).

**Table 4:** Results from docking modeling with HADDOCK.

No	Peptide	HADDOCK Score	Cluster	RMSD (Å)	Z-Score
1	MQML	−52.7 +/− 1.7	2	0.7 +/− 0.1	−1.4
2	AAPNF	−53.5 +/− 10.4	7	1.0 +/− 0.1	−1.8
3	PPPPV	−62.2 +/− 4.8	7	0.5 +/− 0.1	−2.4
4	YPPPT	−65.5 +/− 1.9	4	0.9 +/− 0.1	−1.8
5	PMNPPK	−66.0 +/− 2.2	4	0.2 +/− 0.1	−2.2
6	PPPS	−67.6 +/− 1.9	2	0.3 +/− 0.2	−1.9
7	PMIPG	−72.8 +/− 0.5	2	0.3 +/− 0.2	−1.9
8	AWMIYT	−82.2 +/− 2.6	1	0.6 +/− 0.1	−1
9	AAWMIY	−89.4 +/− 1.9	1	0.5 +/− 0.2	−1.6
10	VNPYKWL	−90.4 +/− 4.6	1	0.2 +/− 0.1	−2.5
11	AMYF	−92.9 +/− 2.0	4	0.7 +/− 0.0	−1.7
12	MVWH	−97.0 +/− 1.2	13	0.1 +/− 0.1	−2

RMSD is an abbreviation of ‘root-mean-square deviation’ from the overall lowest-energy structure.

**Table 5:** Results from PRODIGY.

Peptides	Activity	Binding Affinity ΔG (kcal mol^−1^)	Kd (M) at 25 °C
VNPYKWL	ACE-I	−9.6	8.80 × 10^−8^
PMNPPK	ACE-I	−9.9	5.60 × 10^−8^
PPPPV	ACE-I	−12.3	9.30 × 10^−10^
PMIPG	ACE-I	−10.6	1.60 × 10^−8^
YPPPT	ACE-I	−10.4	2.20 × 10^−8^
AAPNF	ACE-I	−8.5	5.60 × 10^−7^
AMYF	ACE-I	−10.9	1.00 × 10^−8^
MVWH	ACE-I	−11	8.00 × 10^−9^
PPPS	ACE-I	−8.8	3.60 × 10^−7^
AWMIYT	DPPIV-I	−9.7	7.60 × 10^−8^
AAWMIY	DPPIV-I	−9.1	2.20 × 10^−7^
MQML	DPPIV-I	−8.6	4.80 × 10^−7^

ACE-I and DPPIV-I in the activity column represent ACE-inhibitory and DPPIV-inhibitory activities, respectively.

**Table 6:** ACE-inhibitory peptide–residue interactions.

ACE-Inhibitory Peptide	Interacting Residue and Position	Active Site Pocket	Interaction Type
VNPYKWL	Glu 162	S1′	HA, SB
	Glu 376		HA, SB
	Asp 377		SB
	Thr 372		HA
	Cys 370		HA
	Asn 285		HA
YPPPT	Lys 511	S2	HD
	Thr 166		HD
	Asn 374		HA, HD
PPPPV	His 353	S2	HA
	Glu 162	S1′	HA, SB
	Asn 285		HD
	Thr 302		HD
PMIPG	Glu 376		SB
	Asn 285		HA
	Ser 284		HA
	Lys 449		HD
AAPNF	Glu 376		HA
	Asn 374		HD
	Ala 354	S1	HD
	Glu 384	S1	SB
	Tyr 523	S1	HA
PMNPPK	Lys 449		HD
	Glu 376		HD, SB
	Asp 453		HA, SB
	Thr 282		HA
	Ser 284		HA
MVWH	Gln 281	S2	HD
	Asp 377		HA, HA, SB
	Lys 511	S2	HD
	His 383	Tetra-coordinated Zn	Pi
	Glu 384	S1	HA
	His 353	S2	Pi
AMYF	Asp 453		HA
	His 353	S2	HD
PPPS	Glu 384	S1	HA, SB
	Glu 411	Tetra-coordinated Zn	SB
	Tyr 523	S1	HA
	Ala 354	S1	HD

Notes: HA, HD, SB, and Pi are abbreviations for hydrogen bonds where the receptor residue is the acceptor (HA), hydrogen bonds where the receptor residue is the donor (HD), a salt bridge interaction (SB), and a Pi-Pi stacking interaction, respectively.

**Table 7:** DPPIV-inhibitory peptide residue interactions.

DPPIV-Inhibitory Peptide	Interacting Residue	Active Site Pocket	Interaction Type
AAWMIY	Glu 206	S2	HA
	Glu 205	S2	HA
	Ser 209	S3	HA
	Arg 125	S2	HD, Pi-c
	Trp 629		Pi
	Tyr 752		HA
AWMIYT	Arg 125	S2	Pi-c, Pi-c
	Glu 206	S2	HA
	Ser 209	S3	HA
	Tyr 585		HD, Pi
	Arg 356		HD
	Phe 357		HD, Pi
	Arg 358	S3	HA
MQML	His 126		HD
	Arg 125	S2	HD
	Ser 209	S3	HD
	Arg 358	S3	HD

HA, HD, Pi, and Pi-c are abbreviations for hydrogen bonds where the enzyme residue is the acceptor, hydrogen bonds where the enzyme residue is the donor, Pi-Pi stacking, and Pi–cation interactions, respectively.

**Table 8:** Absorption, distribution, and toxicity of twelve different peptides.

Peptide	Absorption		Distribution		Toxicity	
	^a^ MDCK	^b^ HIA	^c^ VD (L/Kg)	^d^ PPB (%)	^e^ HHT	^f^ Ames
VNPYKWL	3.5 × 10^−5^	Low	0.426	31.35	Yes	No
PMNPPK	4.9 × 10^−6^	Low	0.617	19	Yes	No
PPPPV	3.4 × 10^−6^	Low	0.425	21.67	Yes	No
AAPNF	6.4 × 10^−4^	Low	0.283	8.31	No	No
YPPPT	1.5 × 10^−4^	Low	0.369	33.84	Yes	No
PMIPG	1.7 × 10^−6^	Low	0.418	12.11	Yes	No
AMYF	4.4 × 10^−6^	Low	0.262	36	Yes	No
PPPS	6.2 × 10^−5^	Low	0.422	14.85	Yes	No
MVWH	1.7 × 10^−6^	High	0.318	19.86	No	No
AAWMIY	2 × 10^−6^	High	0.331	65.25	Yes	No
AWMIYT	2.2 × 10^−6^	High	0.366	62.72	Yes	No
MQML	7.1 × 10^−6^	High	0.465	12.86	No	No

^a^ MDCK: Madin–Darby canine kidney; ^b^ HIA: human intestinal absorption; ^c^ VD: volume distribution; ^d^ PPB: plasma protein binding; ^e^ HHT: human hepatotoxicity; ^f^ Ames: genotoxicity assay.

**Table 9:** The physicochemical and physiochemical properties of the identified peptides.

Peptide	^a^ MW (<500 Da)	^b^ HBAs (≤10)	^c^ HBDs (≤5)	^d^ logP (≤5)	^e^ TPSA (≤140)	^f^ RO5 Violation
VNPYKWL	918.5	20	14	0.417	334.26	4
PMNPPK	682.35	16	9	−3.067	246.36	4
PPPPV	505.29	11	3	−2.066	139.36	2
AAPNF	518.25	13	8	−2.042	214.02	3
YPPPT	573.28	13	6	−1.391	193.81	3
PMIPG	513.26	11	5	−1.02	156.94	3
AMYF	530.22	10	7	0.568	170.85	3
PPPS	396.2	10	4	−3.053	139.28	0
MVWH	571.26	12	8	0.342	195.09	3
AAWMIY	753.35	15	10	0.944	244.84	3
AWMIYT	783.36	16	11	0.592	265.07	4
MQML	521.23	11	8	−0.222	193.71	3

^a^ MW: molecular weight; ^b^ HBAs: hydrogen bond acceptors; ^c^ hydrogen bond donors; ^d^ log P: octanol/water partition coefficient; ^e^ TPSA: the polar surface area; ^f^ RO5 violation: number of Lipinski rules violated.

## Data Availability

The original contributions presented in the study are included in the article, further inquiries can be directed to the corresponding author.
